# Telomeres and Telomerase. Ciba Foundation Symposium 211

**DOI:** 10.1038/sj.bjc.6690160

**Published:** 1999-02

**Authors:** M C Bibby

**Affiliations:** Clinical Oncology Unit, University of Bradford, Bradford, West Yorkshire, BD7 1DP, UK

## Abstract

*1999 Cancer Research Campaign*


*British Journal of Cancer* (1999) **79**(5/6), 1003–1004
© 1999 Cancer Research Campaign
Article no. bjoc.1998.0160

### Book Review

### Telomeres and Telomerase. Ciba Foundation Symposium 211

Edited by Wiley, 1997

Telomerase is a ribonucleoprotein that maintains the telomeric ends of chromosomes during replication by adding short stretches of nucleotide repeats using a portion of its integral RNA component as the template. This book is a collection of papers presented at a symposium on telomeres and telomerase held at the Ciba Foundation in February 1997, and it is claimed to be state-of-the-art at that time. The volume is constructed in an interesting way, with individual papers from an international group of experts followed by discussion, but in addition to this there are four general discussion chapters. The first half of the book is concerned exclusively with the use of non-mammalian systems and models to dissect the role of telomerase.

Blackburn and colleagues give a short review of the interaction of telomeres and telomerase. They suggest that altering telomeric DNA rather than simply loss of telomeric DNA can block nuclear division and, because synthesis of the correct telomeric DNA sequence is dependent on the telomerase RNA structure, it may be possible in the future to induce changes in telomerase RNA that will cause it to make deleterious telomeres, representing a possible remedy for cancer or other pathogenic cell proliferation. They also point out the likelihood that in mammalian cells telomere length may be negatively regulated by the Rap1p-like telomere binding factor TRF1, so it is important to consider other factors besides telomerase levels as determinants of telomere length maintenance in human cells.

Cech and Lingner review the DNA end replication problem and discuss ciliate telomerase and the use of ciliate models for understanding the solution to the problem. Hughes et al describe the role of the *EST* genes in yeast telomere replication. They explain that although the gene encoding the RNA subunit of telomerase has been identified in a number of species, identification of the protein components of the enzyme has been much more difficult and they have used a genetic approach to the problem using the yeast *Saccharomyces cerevisiae* as their model system. From a large mutant screen designed to identify new mutants with a telomerase-like defect, they identified 22 mutants that mapped to three genes called *EST1*, *EST2* and *EST3*, as well as a novel *EST*-like mutation in a fourth gene previously identified as *CDC13*. They describe genetic and biochemical analysis that showed *EST1* and *CDC13* encode single-stranded telomeric DNA binding proteins, suggesting that these two proteins may function to mediate access of telomerase to the end of the telomere.

Telomeres were first defined 60 years ago in the fruit fly *Drosophila melanogaster* on the basis of chromosome end protection. Biessmann and colleagues describe the unusual telomere elongation mechanism in *Drosophila*. Long retrotransposons *HeT-A* and *TART* transpose to the ends of chromosomes, instead of short repeats that are synthesized by telomerase. Evidence at the time this chapter was written suggested that, unlike the equivalent terminal repeats in yeast and humans, the presence and length of terminal arrays in *Drosophila* may not be critical for cell cycle progression. The authors believe that, whereas telomerase evolved early in the evolution of eukaryotes, telomere elongation by transposition may be a much more recent mechanism.

The next chapter by Marcand and colleagues constitutes a very readable review of the role of the Rap1p protein in sensing or ‘measuring’ telomere length in yeast. The authors propose a simple negative feedback model for telomere length regulation by Rap1p, the essence of which is the proposition that a threshold number of Rap1 molecules bound at a telomere will generate a signal that blocks the elongation of the telomere by telomerase. This will eventually lead to a loss of terminal repeats and, hence, a loss of Rap1p binding sites. When the number of Rap1p molecules falls below the threshold value, telomerase will be active, leading to repeat tract elongation. The role of two Rap1p interacting factors Rif1p and Rif2p in mediating telomere length regulation are also discussed.

The short account by Guarente on chromatin and ageing in yeast and in mammals seems to pose more questions than it answers, whereas the article by Sydney Shall is an interesting review of what constitutes the limited lifespan of cells in culture.

Harley outlines the telomere hypothesis of ageing and immortalization, i.e. that sufficient telomere loss in one or more chromosomes in normal somatic cells triggers cell senescence whereas reactivation of telomeres is necessary for cell immortalization. Some interesting points are made in the next chapter, which is the General Discussion on whether telomeres are correlative or causative in cellular senescence.

The chapter by Shay and colleagues, which is the first in-depth review on cancer in the volume, describes telomerase assays in diagnosis and prognosis. It is clear from the information presented that telomerase activity can be identified in the vast majority of malignant cancers, but the authors point out that the key clinical challenge is to determine if the level of telomerase activity has diagnostic or prognostic utility. The suggestion is that telomerase activity levels may identify patients that will have either favourable or poor prognostic outcomes, whereas in other instances telomerase activity can distinguish between benign and malignant lesions. However, some questions remain unanswered as telomerase activity has been found in cells adjacent to malignant tumours and telomerase is present in a variety of dividing normal cells.

Blasco and colleagues describe mouse models to study the expression of telomerase in tumours. They used two different transgenic mouse models of multistage tumorigenesis and telomerase activity was detected only in late-stage tumours. The development of a telomerase knock-out mouse is also described, but further results were not available at this time.

Newbold addresses a couple of key questions relating to replicative senescence: (i) do genes exist in the normal human genome whose job it is to repress telomerase in normal human cells and, if so, are they functionally inactive in primary human cancer? and (ii) do rodent cells differ fundamentally from the human equivalent with respect to the somatic control of telomerase activity? He shows evidence to support that normal diploid human somatic cells are resistant to immortalization because of a strongly repressed telomerase activity. Telomerase in hamster cells appears to be much less stringently down-regulated, thus increasing their susceptibility to immortalization and malignant transformation.

In a short article on repair and processing events at DNA ends, Lindahl and colleagues remind the reader that cell nuclei contain several abundant enzymes that bind rapidly and avidly to exposed termini of DNA. These include poly(ADP-ribose) polymerase, DNA-dependent protein kinase, several DNA ligases and excision-repair enzymes. Telomeres are normally shielded from these activities by telomere binding proteins but the authors point out that, if incomplete protection of telomeres occurred, the functions of DNA-specific enzymes would be relevant for processing of telomeres, suggesting possible alternative pathways for telomere propogation in telomerase-negative cells.

The final article in the book by Lansdorp et al demonstrates that even though telomeres shorten with replication and with age the role of telomeres in the biology of human haemopoietic stem cells at this juncture is unclear.

The volume concludes with the final general discussion section discussing telomeres and telomerase in other organisms and with the summary from the chairman of the symposium, Sydney Brenner. It is clear from this volume that the telomerase story is still not straightforward and this volume certainly focuses on many of the complexities. Although an extremely interesting and thought-provoking read, the field is advancing at such a rapid pace it is impossible for such a volume to remain up to date. Nevertheless, I found it to be both informative and stimulating and would recommend it as background reading for anyone who is considering working in the area of telomerase in relation to cancer diagnosis, prognosis or therapy.

*Professor Mike C Bibby*
*Clinical Oncology Unit*
*University of Bradford*
*Bradford*
*West Yorkshire*
*BD7 1DP, UK*

*© Cancer Research Campaign 1999*

